# A pharmacovigilance study of etoposide in the FDA adverse event reporting system (FAERS) database, what does the real world say?

**DOI:** 10.3389/fphar.2023.1259908

**Published:** 2023-10-26

**Authors:** Zhiwei Cui, Feiyan Cheng, Lihui Wang, Fan Zou, Rumeng Pan, Yuhan Tian, Xiyuan Zhang, Jing She, Yidan Zhang, Xinyuan Yang

**Affiliations:** ^1^ Department of Obstetrics and Gynecology, The First Affiliated Hospital of Xi’an Jiaotong University, Xi’an, China; ^2^ Department of Respiratory and Critical Care Medicine, Affiliated Hospital of Zunyi Medical University, Zunyi, China; ^3^ Department of General Medicine, Yanan University Affiliated Hospital, Yan’an, China

**Keywords:** etoposide, real-word analysis, pharmacovigilance, adverse drug event, FAERS

## Abstract

**Introduction:** Etoposide is a broad-spectrum antitumor drug that has been extensively studied in clinical trials. However, limited information is available regarding its real-world adverse reactions. Therefore, this study aimed to assess and evaluate etoposide-related adverse events in a real-world setting by using data mining method on the U.S. Food and Drug Administration Adverse Event Reporting System (FAERS) database.

**Methods:** Through the analysis of 16,134,686 reports in the FAERS database, a total of 9,892 reports of etoposide-related adverse drug events (ADEs) were identified. To determine the significance of these ADEs, various disproportionality analysis algorithms were applied, including the reporting odds ratio (ROR), the proportional reporting ratio (PRR), the Bayesian confidence propagation neural network (BCPNN), and the multi-item gamma Poisson shrinker (MGPS) algorithms.

**Results:** As a result, 478 significant disproportionality preferred terms (PTs) that were identified by all four algorithms were retained. These PTs included commonly reported adverse events such as thrombocytopenia, leukopenia, anemia, stomatitis, and pneumonitis, which align with those documented in the drug’s instructions and previous clinical trials. However, our analysis also uncovered unexpected and significant ADEs, including thrombotic microangiopathy, ototoxicity, second primary malignancy, nephropathy toxic, and ovarian failure. Furthermore, we examined the time-to-onset (TTO) of these ADEs using the Weibull distribution test and found that the median TTO for etoposide-associated ADEs was 10 days (interquartile range [IQR] 2–32 days). The majority of cases occurred within the first month (73.8%) after etoposide administration. Additionally, our analysis revealed specific high-risk signals for males, such as pneumonia and cardiac infarction, while females showed signals for drug resistance and ototoxicity.

**Discussion:** These findings provide valuable insight into the occurrence of ADEs following etoposide initiation, which can potentially support clinical monitoring and risk identification efforts.

## 1 Introduction

Etoposide (VP-16) is a semi-synthetic derivative of the natural antibiotic podophyllotoxin, acting as a potent inhibitor of topoisomerase-II (Cheema et al., 2011). This inhibition leads to DNA strand breaks and the induction of apoptosis, triggering mutagenic and cell death pathways (Meresse et al., 2004; Le and Wang, 2023). Upon entry into the human body, etoposide predominantly binds to serum albumin (93%–98%) and undergoes elimination via the kidneys and biliary tract following glucuronidation (Le and Wang, 2023). The recommended oral dose of etoposide for monotherapy or combination therapy is 100–200 mg/m2/day on days 1–5, or 200 mg/m2/day on days 1, 3, and 5 every 3–4 weeks. Since its approval by the FDA in 1983, etoposide has been widely utilized in the treatment of various solid and hematologic tumors, such as small cell lung cancer, germ cell tumors, and lymphoma (McHugh et al., 2020; Rudin et al., 2020; Jeha et al., 2021; Torka et al., 2023). When combined with other chemotherapeutic agents, it has achieved a remission rate of over 80% (Meresse et al., 2004). In a clinical study involving patients with nonseminomatous germ cell tumors, adjuvant etoposide plus cisplatin for 2 cycles demonstrated prolonged disease-specific and relapse-free survival, along with acceptable toxicity and lower drug costs (McHugh et al., 2020).

Adverse drug events (ADEs) are crucial concerns in modern healthcare as they have a significant impact on patient safety, treatment outcomes, and overall public health (Montané and Santesmases, 2020). Given the outstanding efficacy and widespread use of etoposide in the treatment of tumors, it is important to understand its adverse effects to improve patient care (Edwards and Aronson, 2000). Common adverse reactions reported in association with etoposide dosing include myelosuppression, gastrointestinal toxicity, and hypersensitivity reactions (Henwood and Brogden, 1990; Zhu et al., 2016). However, unknown adverse reactions of etoposide are expected to be identified in post-marketing studies due to the limitations of clinical trials, such as restricted populations, limited follow-up time, and complications (Yan et al., 2022; Javed and Kumar, 2023). Therefore, searching for potential ADEs of etoposide through post-marketing surveillance using data mining algorithms is highly warranted.

The pharmacovigilance of drugs relies on the identification of statistical signals derived from diverse data sources (Vogel et al., 2020). Signals in pharmacovigilance refer to novel or known connections between adverse events and drugs (Javed and Kumar, 2023). Disproportionality analysis, a widely utilized method, is employed to detect signals using pharmacovigilance databases (Jain et al., 2023). This type of analysis considers the distribution of all drugs and events in the database, calculates statistical associations between drugs and ADEs, and is frequently employed in post-market safety assessments of drugs (Almenoff et al., 2007; Vogel et al., 2020; Sharma et al., 2023). The FDA Adverse Event Reporting System (FAERS) is a publicly accessible database that collects reports of ADEs from healthcare professionals, patients, and drug manufacturers. It serves as a crucial tool for the FDA’s post-marketing safety monitoring of drugs and medical products, and it is one of the largest pharmacovigilance databases worldwide (Meng et al., 2019). Additionally, due to the large sample size of the FAERS database, data mining techniques possess sufficient statistical power to detect rare adverse reactions that are challenging to identify in traditional epidemiological studies (Duggirala et al., 2016; Jiao et al., 2020; Sharma and Kumar, 2022). Given that etoposide-related adverse reaction reports primarily originate from clinical trials, with a focus on specific organ systems, we utilized the FAERS database to conduct disproportionality analyses. This assessment aimed to evaluate the long-term safety of etoposide through post-marketing surveillance, providing a comprehensive and valuable reference for its real-world safety.

## 2 Material and methods

### 2.1 Data source and pre-processing

**FIGURE 1:**
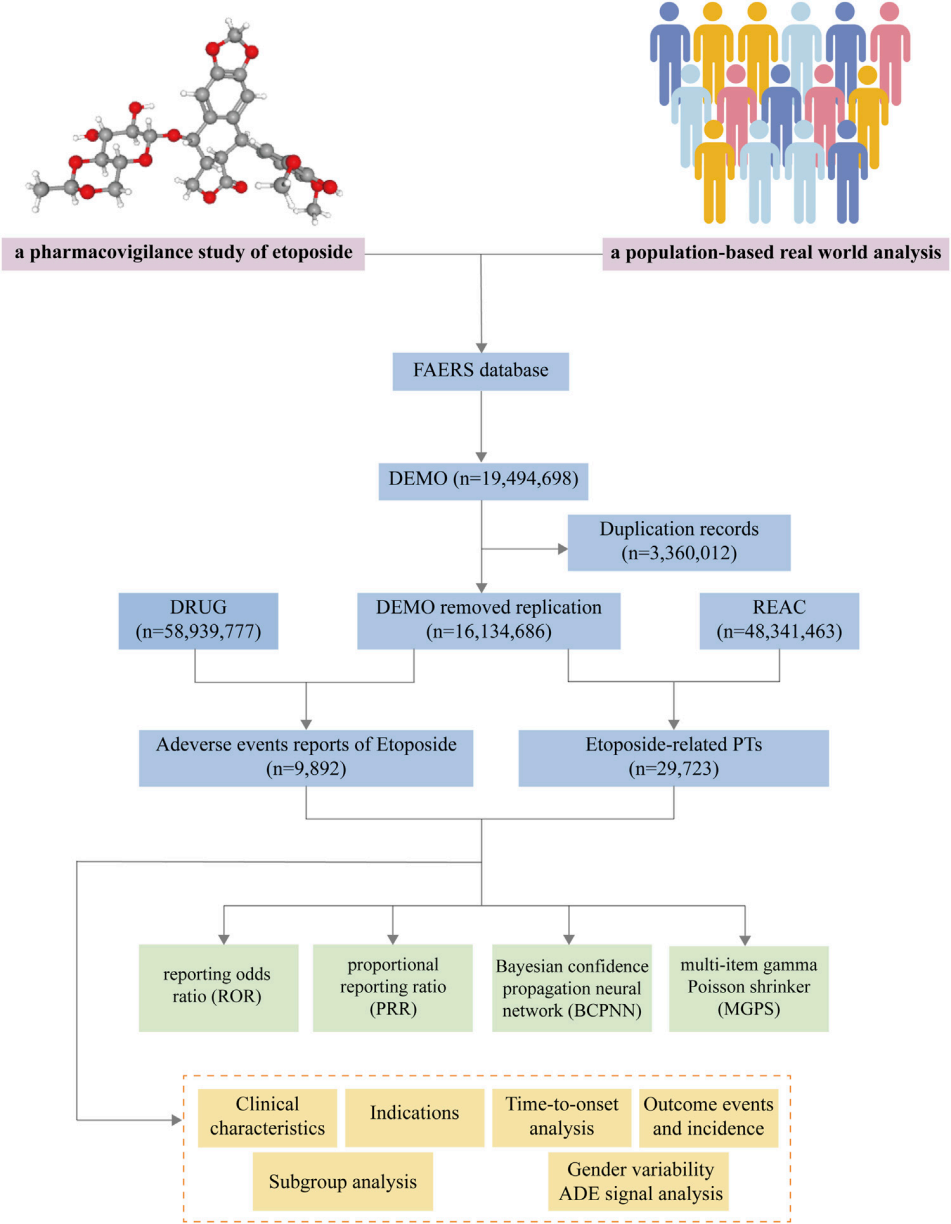
The flow diagram of selecting etoposide-related ADEs from FAERS database.

To systematically evaluate the safety of etoposide in the post-marketing period, we conducted a retrospective pharmacovigilance study using data obtained from the FAERS database. The FAERS database covers data from the first quarter of 2004 to the fourth quarter of 2022 and can be accessed at (https://fis.fda.gov/extensions/FPD-QDE-FAERS/FPD-QDE-FAERS.html). The FAERS data consists of seven datasets: demographic and administrative information (DEMO), drug information (DRUG), adverse drug reaction information (REAC), patient outcomes information (OUCT), reported sources (RPSR), drug therapy start dates and end dates (THER), and indications for drug administration (INDI) (Shu et al., 2022b). We imported all the downloaded data from the FAERS database into SAS software (version 9.4) for further collation and analysis. We acquired a total of 19,494,698 reports. Since the database is updated on a quarterly basis, there will unavoidably be duplication of previous public reports. According to the FDA’s recommendations, we operated deduplication process before statistical analyses, following the criteria: (1) If the CASEIDs were the same, the latest FDA_DT were selected. (2) If the CASEIDs and the FDA_DT were the same, the higher PRIMARYIDs were selected (Shu et al., 2022b). The removing the duplicate records led to a decrease in the number of reports to 16,134,686 (Figure 1). The 3D structure of etoposide is derived from the PubChem (https://pubchem.ncbi.nlm.nih.gov/) (Kim et al., 2023).

### 2.2 Drug identification and adverse events

As FAERS had two variables including DRUGNAME and PROD_AI, both the brand names and common names were employed to recognize records related to etoposide. In this study, “ETOPOSIDE”, “VP-16,” “LASTET,” “TOPOSAR,” “VEPESID,” and “CELLTOP” were used to search. The reported drugs in FAERS were classified into four modalities: PS (primary suspect), SS (second suspect), C (concomitant), and I (interacting). To enhance accuracy, the role code of ADEs was retained only as the PS drug (Zhang et al., 2023). During the period of this research, we identified totally 9892 ADEs reports of etoposide as the PS drug. System Organ Class (SOC) was the highest level of the Medical Dictionary for Regulatory Activities (MedDRA, version 26.0. Available from https://www.meddra.org/) terminology, by which all ADEs in reports were coded of Preferred Terms (PTs) (Tieu and Breder, 2018). Then, 29,723 etoposide-related PTs were screened out (Figure 1). We performed case/non-case analyses to determine whether the ADEs reported for etoposide were statistically significant at the PT and SOC levels compared to other drugs in the complete FAERS database.

Furthermore, the time-to-onset (TTO) of ADEs caused by etoposide were defined as the interval between EVENT_DT (ADEs onset date, in DEMO file) and START_DT (start date of etoposide use, in THER file). Input errors including inaccurate or missing date entries and EVENT_DT earlier than START_DT were eliminated. For exhaustively evaluating the TTO, we incorporated median, quartile, and Weibull shape parameter test in our research (Kinoshita et al., 2020; Shu et al., 2022d). The varying risk incidence increase or decrease of the ADEs over time could be determined and predicted by the Weibull distribution, with scale (α) and shape (*β*) being two parameters used to describe the Weibull distribution shape (Mazhar et al., 2021).

### 2.3 Subgroup analysis

Subgroup analyses were conducted to investigate the association between etoposide dosing and adverse effects in subgroups based on age (<18 [child and adolescent], 18–64 [adult], and >64 [elder]), gender (male and female), weight (<80 kg, 80–100 kg, and >100 kg), and reporting person (consumer and health professional).

### 2.4 Data mining algorithm and statistical analysis

Disproportionality analysis is primarily used as a tool for hypothesizing possible causal relationships between drugs and adverse events. It is based on comparing the observed and expected number of reports for each specific combination of drug and adverse event (Montastruc et al., 2011; Caster et al., 2020; Hu et al., 2020; Sharma et al., 2023). Consequently, in our research, we conducted a disproportionality analysis to determine the potential correlation between etoposide and all ADEs. Considering that separate methods of detecting signals may be insufficient, the four algorithms including reporting odds ratio (ROR), the proportional reporting ratio (PRR), the Bayesian confidence propagation neural network (BCPNN), and the multi-item gamma Poisson shrinker (MGPS) were implied (Lindquist et al., 2000; van Puijenbroek et al., 2002; Shao et al., 2021; Zhou et al., 2021). PRR and ROR are frequencyist (non-Bayesian), while BCPNN and MGPS are Bayesian (Sakaeda et al., 2013). Information Components (IC) are used in the tool BCPNN to measure disproportionality (Hauben and Zhou, 2003; Bate, 2007). MGPS analysis is a well-established technique for reducing the rate of false-positive reports by applying a Bayesian shrinkage estimator to the observed/expected ratio to give smaller risk estimates with narrower confidence intervals, even if the event counts are small (Napoli et al., 2014; Trippe et al., 2017). The two Bayesian methods (BCPNN and MGPS) were considered useful because each detected unique signal even when there were few reports of ADE for a particular drug (Nomura et al., 2015). Overall, the higher the value of the four parameters, the stronger the signal value. The specific formulas and the criteria of positive safety signal detection of the four algorithms were shown in Table 1. Only the signals that had at a minimum of three targeted drug ADEs records were counted. To assure the reliability of the results, we selected ADEs signals that satisfy the above four algorithm criteria simultaneously for the study (Sakaeda et al., 2013; Yin et al., 2022). We also excluded the indications for etoposide from the ADEs to avoid unclear presentation (Tang et al., 2022). The drug label of etoposide was obtained from the DailyMed (https://dailymed.nlm.nih.gov/dailymed/index.cfm)(Yao et al., 2017), and Summary of Product Characteristics (SmPC) (https://www.ema.europa.eu/en/glossary/summary-product-characteristics) (Nezvalova-Henriksen et al., 2023). Novelty/unexpectedness signal is defined as any significant ADEs detected without being outlined in the drug label (Shu et al., 2022a). All processing of data and statistical analyses were carried out using SAS 9.4, Microsoft EXCEL 2019, and R (version 4.2.1).

**TABLE 1:** Four major algorithms used to assess potential associations between etoposide and ADEs. a, Number of reports that contain both targeted drug and targeted drug adverse reactions; b, Number of reports of other drug adverse reactions that contain the targeted drug; c, Number of reports of targeted drug adverse reactions that contain other drugs; d, Number of reports that contain other drugs and other drug adverse reactions. 95% CI, 95% confidence interval; N, the number of reports; χ2, chi-squared; IC, information component; IC025, the lower limit of 95% CI of the IC; E (IC), the IC expectations; V(IC), the variance of IC; EBGM, empirical Bayesian geometric mean; EBGM05, the lower limit of 95% CI of EBGM.

Algorithms	Equation	Criteria
ROR	ROR = (ad/bc)	lower limit of 95% CI > 1
	95%CI = eln (ROR)±1.96 (1/a+1/b+1/c+1/d)^0.5	
PRR	PRR = a (c+d)/c/(a+b)	N ≥ 3 PRR≥2, χ2≥4, N ≥ 3
	χ2 = [(ad-bc)^2](a+b+c+d)/[(a+b)(c+d)(a+c)(b+d)]	
BCPNN	IC = log2a (a+b+c+d)/((a+c)(a+b))	IC025 > 0
	95%CI = E (IC) ± 2V(IC)^0.5	
MGPS	EBGM = a (a+b+c+d)/(a+c)/(a+b)	EBGM05 > 2
	95%CI = eln (EBGM)±1.96 (1/a+1/b+1/c+1/d)^0.5	

## 3 Results

### 3.1 Annual distribution of etoposide-related ADE reports

**FIGURE2:**
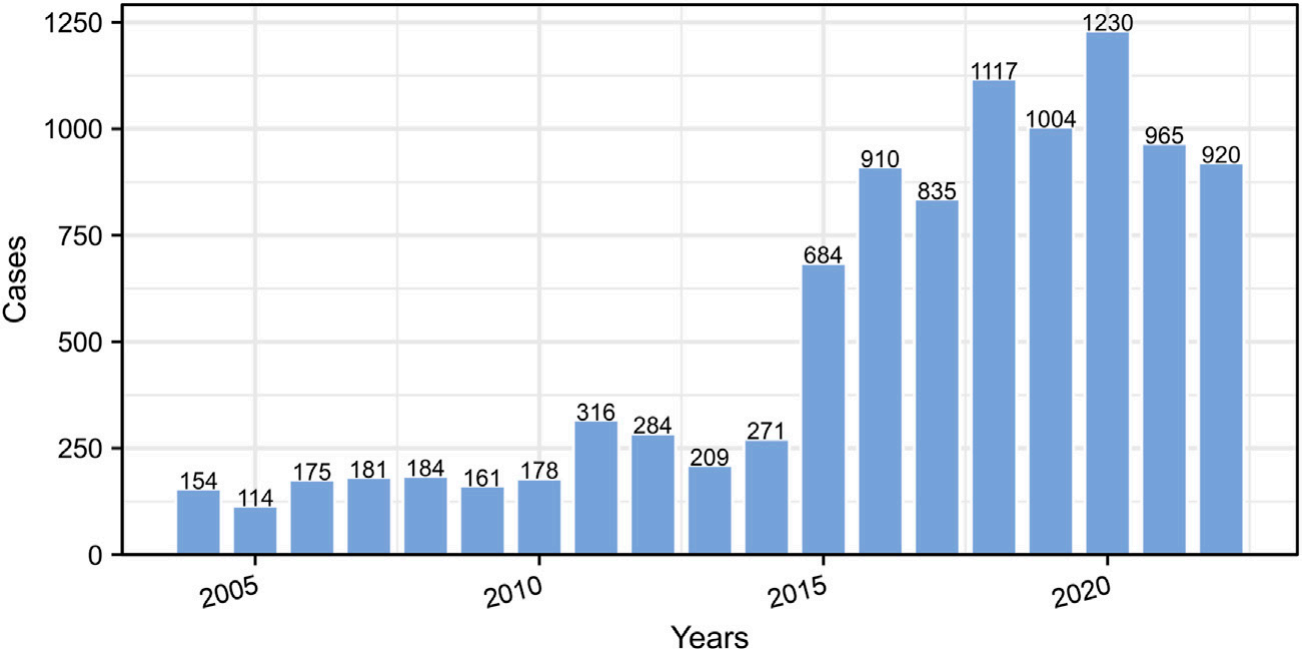
The annual distribution of etoposide-related ADE reports from 2004 to 2022.

According to the data from the FAERS database, there were a total of 9892 ADEs reports for etoposide between January 2004 and December 2022. From an overall perspective, the number of ADE reports has been increasing over the years, as depicted in Figure 2. The lowest and highest number of reports were observed in 2005 (114 reports) and 2020 (1230 reports), respectively. Notably, there was a substantial increase in the number of reports in 2015. More detailed information on the annual distribution can be found in Figure 2.

### 3.2 General characteristics in the real-world population

Table 2 displayed the population characteristics of the ADEs reports associated with etoposide. It is notable that there were more male patients reported (49.6%) compared to female patients (34.3%), potentially due to specific indications of the drug such as testicular cancer. The reported proportions of body weight in the categories of <80 kg, 80–100 kg, and >100 kg was 15.7%, 4.5%, and 1.3%, respectively. A higher occurrence of etoposide-related ADEs was observed in young (22.5%) and middle-aged patients (39.4%) compared to elderly patients (14.1%).

**TABLE 2:** Clinical characteristics of reports with etoposide from the FAERS Database (January 2004–December 2022).

Characteristics	Case number, n	Case proportion, %
**Number of events**	9892	
**Gender**		
Female	3392	34.3
Male	4903	49.6
Unknown	1597	16.1
**Weight (kg)**		
<80	1553	15.7
80–100	449	4.5
>100	127	1.3
Unknown	7763	78.5
**Age (years)**		
<18	2228	22.5
18–64	3893	39.4
>64	1399	14.1
Unknown	2372	24.0
**Reported Countries (top five)**		
America	2694	27.2
France	1100	11.1
Japan	912	9.2
Canada	858	8.7
Italy	494	5.0
**Reported Person**		
Health professional	8779	88.7
Consumer	608	6.2
Unknown	505	5.1
**Serious Outcomes**		
Death (DE)	1937	15.5
Life-threatening (LF)	1041	8.3
Hospitalization (HO)	3065	24.6
Disability (DS)	133	1.1
Other serious outcomes	6308	50.5
**Indications (top five)**		
Product used for unknown indication	661	6.7
Acute myeloid leukaemia	456	4.6
Small cell lung cancer	370	3.7
Hodgkin’s disease	360	3.6
Acute lymphocytic leukaemia	344	3.5

The majority of ADE reports were from the United States (27.2%), followed by France (11.1%), Japan (9.2%), Canada (8.7%), and Italy (5.0%). Interestingly, health professionals accounted for the highest proportion (88.7%) of these reports. Among the serious outcomes reported, 50.5% were classified as “other serious outcomes,” while the most frequently reported serious outcome was hospitalization (24.6%). Additionally, the percentages of death and life-threatening outcomes were 15.5% and 8.3%, respectively. These outcomes may be more closely associated with the progression of the underlying tumor.

The top five indications for etoposide use included cases where the product was used for an unknown indication (6.7%), acute myeloid leukemia (4.6%), small cell lung cancer (3.7%), Hodgkin’s disease (3.6%), and acute lymphocytic leukemia (3.5%).

### 3.3 Signals detection at the system organ class level

Table 3 presented the signal strength and number of reports for etoposide at the System Organ Class (SOC) level. Our statistical analysis identified a total of 27 organ systems that were implicated in etoposide-induced ADEs. The SOC that met all four criteria simultaneously and showed significant association with etoposide ADEs was blood and lymphatic system disorders (SOC code: 10005329, 3745 reports).

**TABLE 3:** Signal strength of ADEs of etoposide at the System Organ Class (SOC) level in FAERS database. An asterisk indicates a positive signal value under this algorithm. ROR, reporting odds ratio; CI, confidence interval; PRR, proportional reporting ratio; χ2, chi-squared; IC, information component; EBGM, empirical Bayesian geometric mean.

SOC name	Case number	ROR (95%CI)	PRR (χ2)	IC(IC025)	EBGM(EBGM05)
Blood and lymphatic system disorders	3745	8.53 (8.24–8.83)*	7.58 (21665.64)*	2.92 (1.25)*	7.55 (7.34)*
Infections and infestations	3641	2.57 (2.48–2.66)*	2.37 (3050.51)*	1.25 (-0.42)	2.37 (2.30)*
General disorders and administration site conditions	3106	0.56 (0.54–0.58)	0.60 (985.15)	−0.73 (-2.40)	0.60 (0.58)
Injury, poisoning and procedural complications	2500	0.85 (0.82–0.89)	0.86 (60.59)	−0.21 (-1.88)	0.86 (0.83)
Gastrointestinal disorders	2249	0.87 (0.84–0.91)	0.88 (38.71)	−0.18 (-1.85)	0.88 (0.85)
Respiratory, thoracic and mediastinal disorders	2075	1.51 (1.44–1.58)*	1.47 (328.26)	0.56 (-1.11)	1.47 (1.42)
Neoplasms benign, malignant and unspecified (incl cysts and polyps)	1852	2.34 (2.23–2.45)*	2.25 (1327.28)*	1.17 (-0.49)	2.25 (2.17)*
Nervous system disorders	1724	0.65 (0.62–0.68)	0.67 (305.59)	−0.58 (-2.24)	0.67 (0.64)
Investigations	1406	0.74 (0.70–0.78)	0.75 (119.71)	−0.41 (-2.07)	0.75 (0.72)
Cardiac disorders	995	1.24 (1.16–1.32)*	1.23 (43.13)	0.30 (-1.37)	1.23 (1.16)
Vascular disorders	976	1.52 (1.43–1.62)*	1.50 (167.46)	0.59 (-1.08)	1.50 (1.42)
Skin and subcutaneous tissue disorders	856	0.52 (0.49–0.56)	0.54 (361.48)	−0.90 (-2.56)	0.54 (0.51)
Renal and urinary disorders	854	1.47 (1.37–1.57)*	1.46 (124.1)	0.54 (-1.12)	1.46 (1.37)
Metabolism and nutrition disorders	808	1.25 (1.16–1.34)*	1.24 (38.53)	0.31 (-1.36)	1.24 (1.17)
Hepatobiliary disorders	566	2.10 (1.93–2.28)*	2.08 (318.85)*	1.05 (-0.61)	2.08 (1.94)
Musculoskeletal and connective tissue disorders	441	0.27 (0.25–0.30)	0.28 (855.40)	−1.83 (-3.50)	0.28 (0.26)
Immune system disorders	441	1.36 (1.24–1.50)*	1.36 (41.95)	0.44 (-1.23)	1.36 (1.25)
Psychiatric disorders	292	0.16 (0.14–0.18)	0.17 (1246.38)	−2.55 (-4.21)	0.17 (0.16)
Surgical and medical procedures	231	0.61 (0.53–0.69)	0.61 (58.82)	−0.72 (-2.38)	0.61 (0.55)
Eye disorders	217	0.37 (0.32–0.42)	0.37 (235.75)	−1.43 (-3.10)	0.37 (0.33)
Ear and labyrinth disorders	184	1.42 (1.23–1.64)*	1.42 (22.70)	0.50 (-1.16)	1.42 (1.26)
Reproductive system and breast disorders	149	0.54 (0.46–0.63)	0.54 (59.52)	−0.89 (-2.56)	0.54 (0.47)
Congenital, familial and genetic disorders	143	1.53 (1.29–1.80)*	1.52 (25.73)	0.61 (-1.06)	1.52 (1.33)
Endocrine disorders	114	1.54 (1.28–1.85)*	1.54 (21.63)	0.62 (-1.04)	1.54 (1.32)
Pregnancy, puerperium and perinatal conditions	96	0.72 (0.59–0.89)	0.73 (10.00)	−0.46 (-2.13)	0.73 (0.61)
Product issues	48	0.11 (0.08–0.14)	0.11 (363.25)	−3.22 (-4.89)	0.11 (0.08)
Social circumstances	14	0.10 (0.06–0.17)	0.10 (112.74)	−3.31 (-4.97)	0.10 (0.07)

Additionally, other significant SOCs that met three criteria at the same time included infections and infestations (SOC code: 10021881, 3641 reports), and neoplasms benign, malignant, and unspecified (including cysts and polyps) (SOC code: 10029104, 1852 reports). Furthermore, there were several other significant SOCs that met at least one of the criteria. These included respiratory, thoracic, and mediastinal disorders (SOC code: 10038738, 2075 reports), cardiac disorders (SOC code: 10007541, 995 reports), vascular disorders (SOC code: 10047065, 976 reports), renal and urinary disorders (SOC code: 10038359, 854 reports), metabolism and nutrition disorders (SOC code: 10027433, 808 reports), hepatobiliary disorders (SOC code: 10019805, 566 reports), immune system disorders (SOC code: 10021428, 441 reports), ear and labyrinth disorders (SOC code: 10013993, 184 reports), congenital, familial, genetic disorders (SOC code: 10010331, 143 reports), and endocrine disorders (SOC code: 10014698, 114 reports).

### 3.4 Signals detection at the preferred terms level

A total of 478 etoposide-induced ADEs that covering 26 SOCs at the PT level were detected after compliance with all four algorithms simultaneously. The full results were listed in Supplementary Table S1. We then ranked all the PTs with ADEs case number exceeding 30 (a>30) according to the value of EBGM05 (the most stringent algorithm) from largest to smallest, and selected a total of 68 ADEs that met the screening criteria (Sakaeda et al., 2013). They were grouped by SOC and the result was shown in Table 4.

**TABLE 4:** Signal strength of ADEs of etoposide at the Preferred terms (PTs) level in FAERS database. Asterisks indicate new and significant signals of etoposide-associated ADEs from FAERS database. ROR, reporting odds ratio; CI, confidence interval; PRR, proportional reporting ratio; χ2, chi-squared; IC, information component; EBGM, empirical Bayesian geometric mean.

SOC name	Preferred terms (PTs)	Case numbers	ROR(95%Cl)	PRR	χ2	IC (IC025)	EBGM (EBGM05)
Blood and lymphatic system disorders	Febrile bone marrow aplasia*	80	42.49 (34.03–53.07)	42.38	3150.61	5.37 (3.70)	41.33 (34.32)
Blood and lymphatic system disorders	Febrile neutropenia	874	30.34 (28.35–32.47)	29.48	23639.47	4.86 (3.19)	28.97 (27.37)
Blood and lymphatic system disorders	Bone marrow failure*	248	22.15 (19.53–25.12)	21.98	4901.18	4.44 (2.77)	21.70 (19.53)
Blood and lymphatic system disorders	Haematotoxicity	87	23.16 (18.74–28.63)	23.10	1813.81	4.51 (2.84)	22.79 (19.09)
Blood and lymphatic system disorders	Aplastic anaemia	60	23.97 (18.57–30.94)	23.93	1299.08	4.56 (2.89)	23.59 (19.06)
Blood and lymphatic system disorders	Pancytopenia	386	14.89 (13.46–16.47)	14.71	4893.66	3.87 (2.20)	14.59 (13.41)
Blood and lymphatic system disorders	Neutropenia	561	9.43 (8.67–10.25)	9.27	4123.11	3.21 (1.54)	9.22 (8.60)
Blood and lymphatic system disorders	Thrombocytopenia	417	7.96 (7.23–8.77)	7.86	2490.27	2.97 (1.30)	7.83 (7.22)
Blood and lymphatic system disorders	Disseminated intravascular coagulation*	62	8.42 (6.56–10.80)	8.40	402.2	3.06 (1.40)	8.36 (6.78)
Blood and lymphatic system disorders	Leukopenia	176	7.36 (6.34–8.54)	7.32	957.31	2.87 (1.20)	7.29 (6.44)
Blood and lymphatic system disorders	Thrombotic microangiopathy*	35	8.41 (6.04–11.73)	8.41	227.22	3.06 (1.40)	8.37 (6.34)
Blood and lymphatic system disorders	Lymphopenia	50	7.66 (5.80–10.11)	7.64	287.48	2.93 (1.26)	7.61 (6.03)
Blood and lymphatic system disorders	Cytopenia	34	7.84 (5.59–10.98)	7.83	201.58	2.96 (1.30)	7.80 (5.88)
Blood and lymphatic system disorders	Myelosuppression	47	6.32 (4.74–8.41)	6.31	209.16	2.65 (0.99)	6.29 (4.95)
Blood and lymphatic system disorders	Agranulocytosis	45	5.60 (4.18–7.51)	5.59	169.21	2.48 (0.81)	5.58 (4.37)
Blood and lymphatic system disorders	Anaemia	345	3.64 (3.27–4.04)	3.61	650.42	1.85 (0.18)	3.60 (3.29)
Cardiac disorders	Cardiotoxicity*	73	19.62 (15.57–24.71)	19.57	1271.21	4.27 (2.61)	19.35 (15.95)
Cardiac disorders	Acute myocardial infarction	53	3.37 (2.58–4.42)	3.37	88.13	1.75 (0.08)	3.36 (2.68)
Congenital, familial and genetic disorders	Aplasia	61	52.62 (40.77–67.92)	52.52	2986.25	5.67 (4.00)	50.90 (41.11)
Ear and labyrinth disorders	Ototoxicity*	37	44.18 (31.86–61.25)	44.12	1518.22	5.43 (3.76)	42.98 (32.70)
Ear and labyrinth disorders	Deafness*	51	4.10 (3.12–5.40)	4.10	119.16	2.03 (0.37)	4.09 (3.25)
Gastrointestinal disorders	Neutropenic colitis*	42	51.05 (37.54–69.42)	50.98	1995.59	5.63 (3.96)	49.46 (38.25)
Gastrointestinal disorders	Oesophagitis	45	8.31 (6.20–11.14)	8.30	287.4	3.05 (1.38)	8.26 (6.46)
Gastrointestinal disorders	Colitis*	72	4.23 (3.36–5.34)	4.23	176.96	2.08 (0.41)	4.22 (3.47)
Gastrointestinal disorders	Stomatitis	109	3.83 (3.17–4.62)	3.82	226.59	1.93 (0.27)	3.81 (3.26)
General disorders and administration site conditions	Mucosal inflammation	256	20.84 (18.41–23.58)	20.67	4732.89	4.35 (2.69)	20.42 (18.41)
General disorders and administration site conditions	Multiple organ dysfunction syndrome*	165	7.53 (6.46–8.78)	7.50	925.24	2.90 (1.23)	7.47 (6.57)
General disorders and administration site conditions	Drug resistance*	46	3.92 (2.94–5.24)	3.92	99.84	1.97 (0.30)	3.91 (3.07)
Hepatobiliary disorders	Venoocclusive liver disease*	107	48.75 (40.21–59.11)	48.58	4841.96	5.56 (3.89)	47.20 (40.17)
Hepatobiliary disorders	Hepatotoxicity	41	4.00 (2.94–5.44)	4.00	91.88	2.00 (0.33)	3.99 (3.09)
Hepatobiliary disorders	Hepatic failure*	52	3.39 (2.58–4.46)	3.39	87.43	1.76 (0.09)	3.38 (2.69)
Immune system disorders	Haemophagocytic lymphohistiocytosis*	80	19.91 (15.97–24.83)	19.86	1415.83	4.30 (2.63)	19.63 (16.32)
Immune system disorders	Anaphylactic reaction	94	3.83 (3.13–4.70)	3.82	195.81	1.93 (0.27)	3.82 (3.22)
Infections and infestations	Neutropenic sepsis*	104	28.74 (23.67–34.90)	28.65	2727.12	4.82 (3.15)	28.17 (23.94)
Infections and infestations	Aspergillus infection*	57	15.77 (12.14–20.47)	15.74	779.22	3.96 (2.30)	15.60 (12.54)
Infections and infestations	Bacteraemia*	69	12.95 (10.21–16.41)	12.92	753.03	3.68 (2.01)	12.83 (10.52)
Infections and infestations	Septic shock*	216	10.82 (9.46–12.38)	10.75	1899.2	3.42 (1.75)	10.69 (9.55)
Infections and infestations	Bronchopulmonary aspergillosis*	36	10.13 (7.30–14.06)	10.12	294.15	3.33 (1.66)	10.07 (7.65)
Infections and infestations	Cytomegalovirus infection*	71	8.98 (7.11–11.35)	8.96	499.8	3.16 (1.49)	8.92 (7.34)
Infections and infestations	Pneumocystis jirovecii pneumonia*	51	8.85 (6.72–11.66)	8.84	352.82	3.14 (1.47)	8.80 (6.99)
Infections and infestations	Sepsis*	379	6.97 (6.29–7.71)	6.89	1904.02	2.78 (1.11)	6.87 (6.31)
Infections and infestations	*Clostridium difficile* colitis*	38	7.41 (5.38–10.19)	7.40	209.34	2.88 (1.22)	7.37 (5.64)
Infections and infestations	*Candida* infection*	49	4.94 (3.73–6.54)	4.93	153.24	2.30 (0.63)	4.92 (3.89)
Infections and infestations	Bacterial infection*	39	4.60 (3.36–6.30)	4.59	109.38	2.20 (0.53)	4.58 (3.52)
Infections and infestations	Fungal infection*	62	3.86 (3.01–4.96)	3.86	131.03	1.95 (0.28)	3.85 (3.13)
Infections and infestations	*Clostridium difficile* infection*	32	3.24 (2.29–4.58)	3.24	49.34	1.69 (0.03)	3.23 (2.42)
Injury, poisoning and procedural complications	Infusion related reaction*	161	5.53 (4.74–6.46)	5.51	592.5	2.46 (0.79)	5.49 (4.82)
Injury, poisoning and procedural complications	Off label use	1143	3.47 (3.27–3.69)	3.38	1932.16	1.75 (0.09)	3.37 (3.21)
Investigations	Ejection fraction decreased*	31	4.07 (2.86–5.79)	4.06	71.48	2.02 (0.35)	4.06 (3.02)
Metabolism and nutrition disorders	Tumour lysis syndrome	145	37.61 (31.89–44.36)	37.43	5026.69	5.19 (3.53)	36.61 (31.89)
Neoplasms benign, malignant and unspecified (incl cysts and polyps)	Myelodysplastic syndrome*	189	26.49 (22.94–30.60)	26.33	4533.37	4.70 (3.03)	25.93 (22.98)
Neoplasms benign, malignant and unspecified (incl cysts and polyps)	Second primary malignancy*	43	10.32 (7.64–13.93)	10.30	358.96	3.36 (1.69)	10.24 (7.97)
Neoplasms benign, malignant and unspecified (incl cysts and polyps)	Malignant neoplasm progression*	203	4.45 (3.87–5.11)	4.42	537.25	2.14 (0.48)	4.41 (3.93)
Nervous system disorders	Posterior reversible encephalopathy syndrome*	102	21.69 (17.84–26.38)	21.62	1980.34	4.42 (2.75)	21.35 (18.13)
Nervous system disorders	Peripheral sensory neuropathy	33	12.22 (8.67–17.21)	12.20	336.95	3.60 (1.93)	12.12 (9.10)
Nervous system disorders	Encephalopathy*	102	8.98 (7.39–10.91)	8.95	716.68	3.15 (1.49)	8.91 (7.57)
Nervous system disorders	Neurotoxicity	32	4.23 (2.99–5.99)	4.23	78.76	2.08 (0.41)	4.22 (3.16)
Renal and urinary disorders	Nephropathy toxic*	72	14.88 (11.79–18.77)	14.84	921.35	3.88 (2.21)	14.72 (12.12)
Reproductive system and breast disorders	Ovarian failure*	43	179.93 (131.29–246.60)	179.67	6879.7	7.34 (5.67)	161.89 (124.36)
Respiratory, thoracic and mediastinal disorders	Pulmonary toxicity	37	12.61 (9.12–17.43)	12.59	391.94	3.64 (1.98)	12.51 (9.54)
Respiratory, thoracic and mediastinal disorders	Acute respiratory distress syndrome*	77	8.88 (7.10–11.11)	8.86	534.06	3.14 (1.47)	8.82 (7.31)
Respiratory, thoracic and mediastinal disorders	Pulmonary haemorrhage*	34	8.32 (5.94–11.66)	8.32	217.77	3.05 (1.38)	8.28 (6.24)
Respiratory, thoracic and mediastinal disorders	Pneumonitis	67	5.61 (4.41–7.13)	5.60	252.21	2.48 (0.81)	5.58 (4.57)
Respiratory, thoracic and mediastinal disorders	Respiratory failure*	183	5.02 (4.34–5.80)	4.99	583.1	2.32 (0.65)	4.98 (4.41)
Respiratory, thoracic and mediastinal disorders	Respiratory distress*	70	5.05 (3.99–6.39)	5.04	226.08	2.33 (0.66)	5.03 (4.13)
Respiratory, thoracic and mediastinal disorders	Hypoxia*	68	4.09 (3.22–5.19)	4.08	157.79	2.03 (0.36)	4.07 (3.34)
Surgical and medical procedures	Stem cell transplant	33	28.65 (20.31–40.43)	28.62	864.50	4.81 (3.15)	28.14 (21.10)
Vascular disorders	Venoocclusive disease	41	34.97 (25.66–47.66)	34.93	1322.82	5.10 (3.43)	34.21 (26.41)
Vascular disorders	Flushing	196	3.69 (3.20–4.24)	3.67	380.31	1.87 (0.21)	3.66 (3.26)
Vascular disorders	Cyanosis	34	4.26 (3.04–5.97)	4.26	84.58	2.09 (0.42)	4.25 (3.21)

In our study, some PTs including thrombocytopenia (PT:10043555, case number 417), leukopenia (PT:10024384, case number 176), myelosuppression (PT:10028584, n = 47), febrile neutropenia (PT:10016288, n = 874), anaemia (PT:10002034, case number 345), oesophagitis (PT:10030216, n = 45), stomatitis (PT:10042128, n = 109), hepatotoxicity (PT:10019851, n = 41), peripheral sensory neuropathy (PT:10034620, n = 33), neurotoxicity (PT:10029350, n = 32), and pneumonitis (PT:10035742, n = 67) were complied with warnings in instructions and drug labels. Of particular note, more than 40 unexpected significant ADEs were uncovered in drug labels, including disseminated intravascular coagulation (PT:10013442, n = 62), thrombotic microangiopathy (PT:10043645, n = 35), cardiotoxicity (PT:10048610, n = 73), ototoxicity (PT:10033109, n = 37), deafness (PT:10011878, n = 51), multiple organ dysfunction syndrome (PT:10077361, n = 165), drug resistance (PT:10059866, n = 46), hepatic failure (PT:10019663, n = 52), bacteraemia (PT:10003997, n = 69), sepsis (PT:10040047, n = 379), *clostridium difficile* infection (PT:10054236, n = 32), second primary malignancy (PT:10039801, n = 43), malignant neoplasm progression (PT:10051398, n = 203), encephalopathy (PT:10014625, n = 102), nephropathy toxic (PT:10029155, n = 72), ovarian failure (PT:10033165, n = 43), acute respiratory distress syndrome (PT:10001052, n = 77), respiratory failure (PT:10038695, n = 183), hypoxia (PT:10021143, n = 68), and so on. Furthermore, although there were some PTs with a small number of cases, the signal value intensity was high, such as Erythema ab igne (n = 4, EBGM 722.84 [240.57]), primary hypogonadism (n = 16, EBGM 194.20 [125.44]), hypertensive hydrocephalus (n = 3, EBGM 232.34 [88.55]), genotoxicity (n = 4, EBGM 191.34 [79.92]), renal salt-wasting syndrome (n = 19, EBGM 105.11 [71.23]). This suggested that the occurrence of these ADEs and etoposide administration were also closely related and deserved clinical attention. Supplementary Table S1 provided the EGBM 05 rankings of all the 478 PTs. Supplementary Table S2 summarizes all the adverse reactions mentioned in the DailyMed and SmPC instructions. Supplementary Figures S1A, S1B intersects the positive signals found in this study with the adverse drug reactions mentioned in DailyMed and SmPC. To sum up, the ADEs analysis of real-world data based on the FAERS database could also provide a great reference for the revision of etoposide instructions.

### 3.5 Time-to-onset analysis of etoposide-related ADEs

The onset times of etoposide-related ADEs were extracted and analyzed from the FAERS database. After removing any missing or incorrect onset time reports, a total of 2138 ADEs with available onset times were included in the analysis. The median onset time was found to be 10 days, with an interquartile range (IQR) of 2–32 days.

**FIGURE 3:**
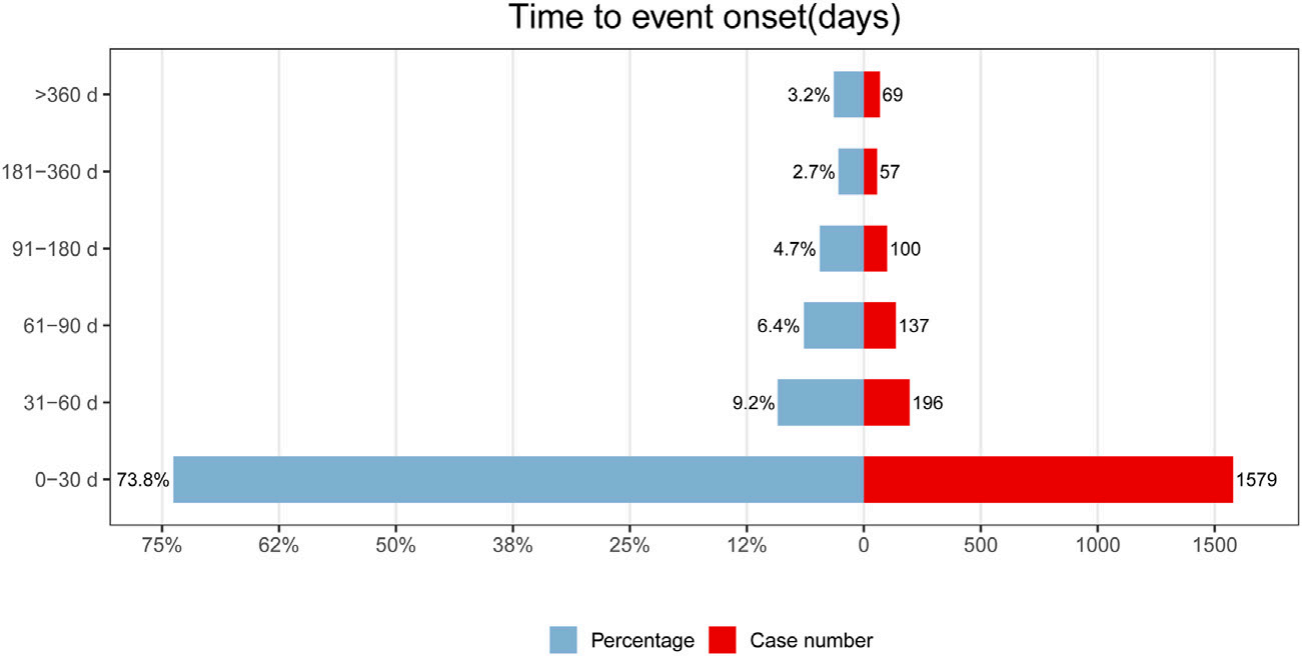
Time-to-onset of etoposide-related ADEs.

Regarding the distribution of ADEs over time, Figure 3 illustrates that the majority of ADEs occurred within the first month after etoposide administration (n = 1579, 73.8%). The number of ADEs decreased with a time delay, with 196 ADEs (9.2%) occurring in the second month and 137 ADEs (6.4%) occurring in the third month. Notably, our data showed that adverse events may still occur after 1 year of etoposide treatment, accounting for 3.2% of cases.

In the evaluation of the Weibull Shape Parameter analysis (Table 5), the shape parameter (*β*) was calculated to be 0.55, and the upper limit of its 95% confidence interval (CI) was 0.57. The value of *β* < 1 suggested that the incidence of ADEs was considered to decrease over time, indicating an early failure type.

**TABLE 5:** Time-to-onset analysis for etoposide-related signals using the Weibull distribution test. n, number of cases with available time-to-onset; IQR, interquartile range; TTO, Time-to-onset. A TTO of 0 days means that the adverse event happens within the day of treatment.

Casesn	TTO (days)		Weibull distribution				Failure type
			Scale parameter		Shape parameter		
	Media (IQR)	Min-Max	α	95% CI	β	95% CI	
2138	10 (2–32)	0–4900	38.56	35.05–42.07	0.55	0.53–0.57	Early failure

### 3.6 Subgroup analysis

**FIGURE 4:**
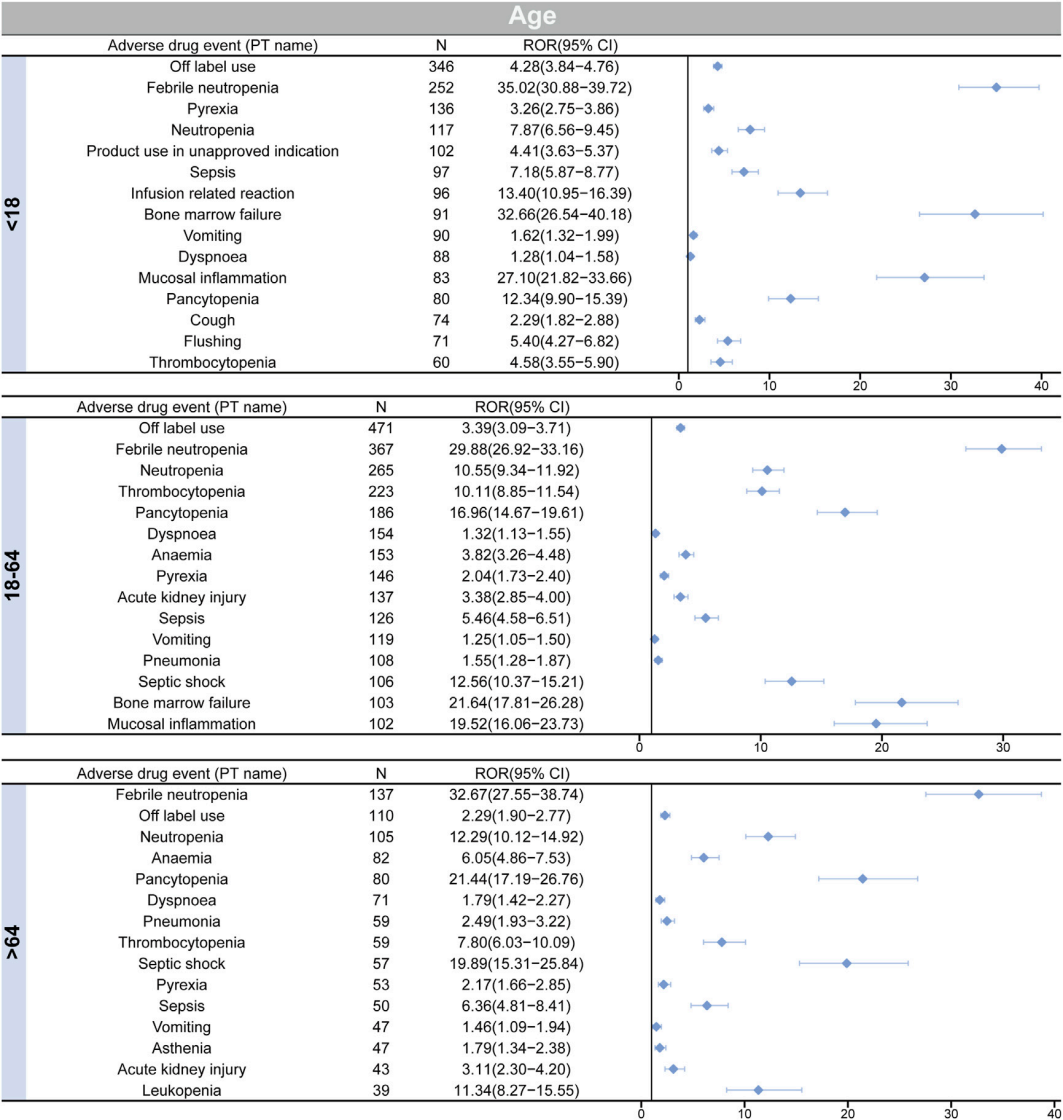
Age-based subgroup analysis of etoposide-related ADEs.

Figure 4 illustrates the findings of the disproportionate analysis stratified by patient age. Among the two subgroups aged <18 and 18–64 years, the highest number of cases were associated with the positive signal of “off-label use.” Conversely, in the >64 age subgroup, “febrile neutropenia” had the highest number of cases. Furthermore, when analyzing the number of top 10 ADEs in each subgroup, it was found that cough and flushing signals were only reported in the subgroup of age <18. On the other hand, acute kidney injury and pneumonia were found to be more common in the other two age subgroups (18–64, and >64).

Likewise, this subgroup disparity in ADEs was also evaluated across weight (Supplementary Figure S2), gender (Supplementary Figure S3), and reporting person (Supplementary Figure S4). These subgroup analyses provide a way to compare signal values between different subgroups, allowing for the identification of similarities and differences. This information is crucial for more detailed clinical management and can help healthcare professionals tailor their approach based on specific subgroup characteristics.

### 3.7 Gender differences in etoposide-related ADEs

**FIGURE 5:**
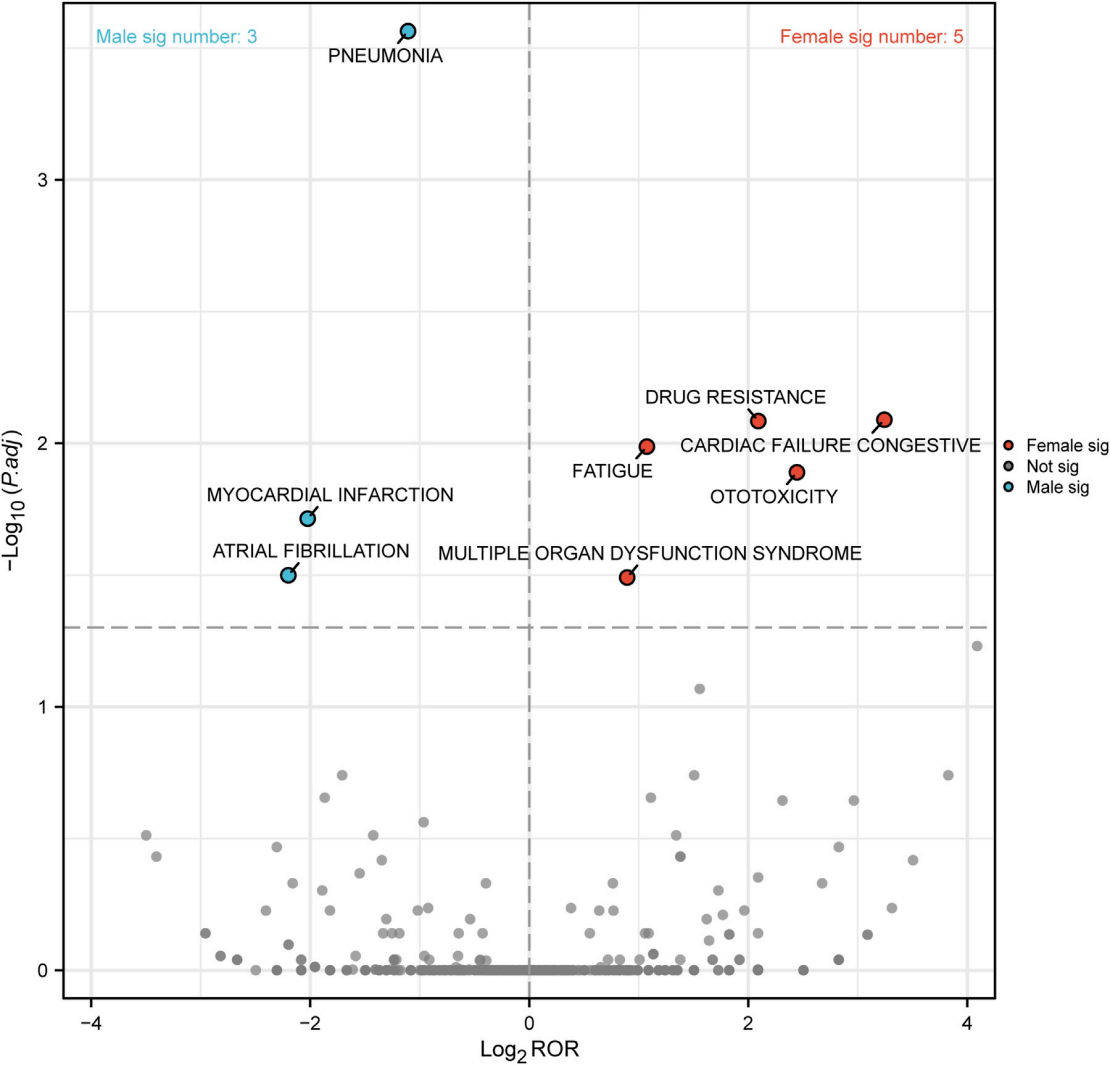
Reporting odds ratios (ROR) with 95% CI for all positive gender-related ADEs. CI, confidence interval; ROR, Reporting odds ratio.

At the PT level, using the ROR algorithm, we identified 58 signals that showed disproportionality in the occurrence of ADEs between males and females. Some of the major ADEs that were more likely to occur in women included cardiac failure congestive, primary hypogonadism, nausea, oesophagitis, death, disease progression, drug resistance, fatigue, multiple organ dysfunction syndrome, hepatic function abnormality, haemophagocytic lymphohistiocytosis, staphylococcal infection, and urinary tract infection. On the other hand, high-risk ADEs in males included leukopenia, thrombocytopenia, pneumonia, hyponatremia, neoplasm progression, peripheral neuropathy, confusional state, acute kidney injury, and pulmonary embolism (Figure 5). You could find all the detailed results in Supplementary Table S3.

**FIGURE 6:**
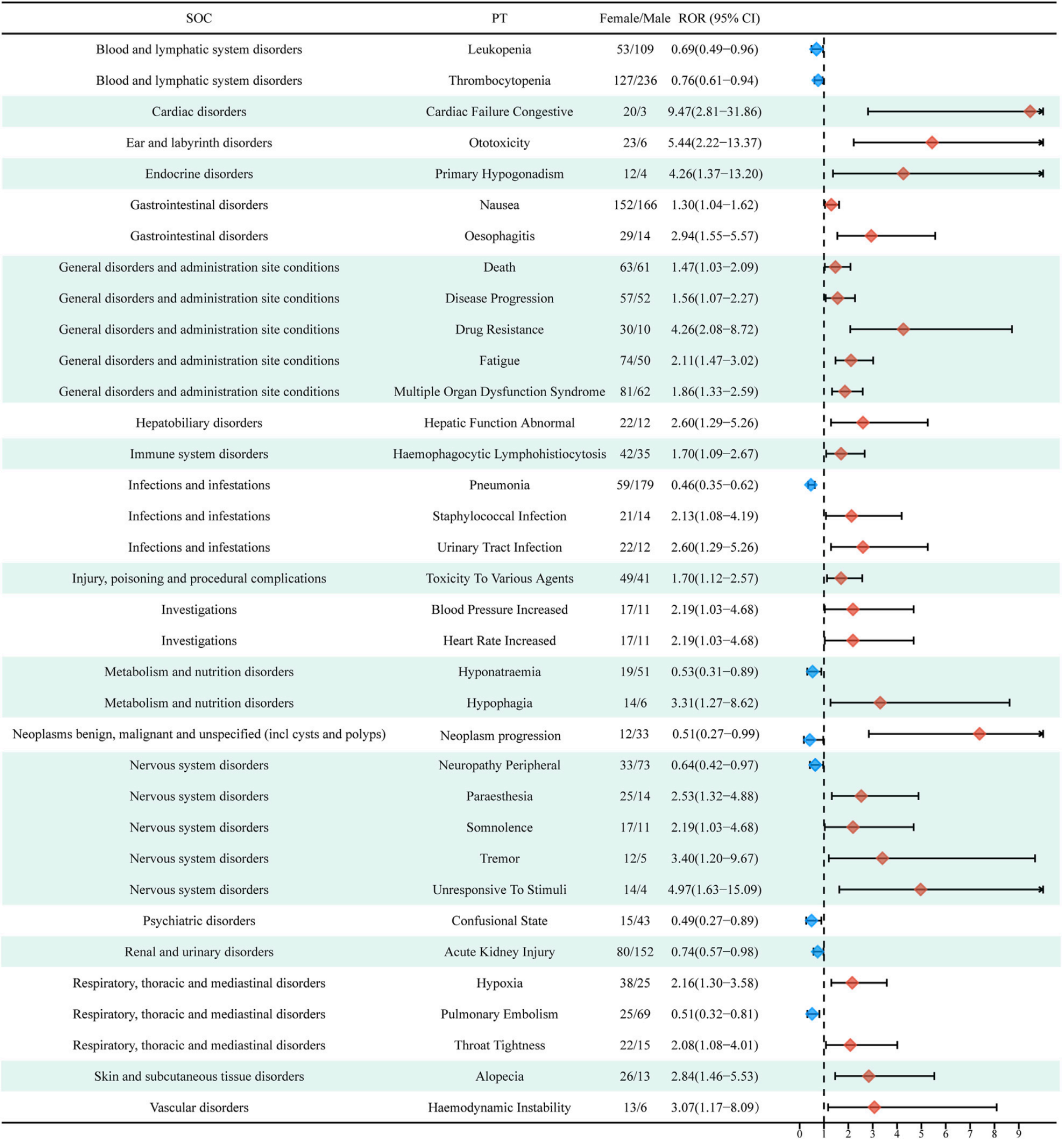
Volcano map of gender difference risk signal for etoposide. ROR, reporting odds ratios; *P*.adj, the *p*-value is adjusted with false discovery rate (FDR) method.

To further differentiate etoposide-related ADEs in terms of gender, we generated a “volcano plot” in Figure 6 to visualize the results. Each point in the plot represented an etoposide-related ADE, and ADEs with significant Log2ROR and -log10 (adjusted *p*-value) were labeled. In males, three significant signals were observed, including pneumonia, myocardial infarction, and atrial fibrillation. In females, five significant signals were found including drug resistance, cardiac failure congestive, fatigue, ototoxicity, and multiple organ dysfunction syndrome (Figure 6).

## 4 Discussion

We conducted a post-marketing pharmacovigilance analysis of etoposide by collecting and evaluating real-world data from the largest sample, with the aim of identifying potential, new adverse reactions to etoposide and analyzing the onset time of adverse reactions as well as gender differences. These findings may help guide updates to the SmPC and provide new evidence for the rational use of etoposide in clinical practice.

### 4.1 Baseline data description

Our study uncovered a yearly increase in the number of reported adverse event reports associated with etoposide, beginning in 2004 and maintaining a relatively high level since 2016. This upward trend suggests not only the effectiveness of etoposide treatment, leading to its increased use in various indications and patient populations, but also emphasizes the importance of analyzing these adverse reactions. Another significant finding was that etoposide-related adverse events occurred more commonly in males (49.6%) compared to females (34.3%). This observation aligns with the higher incidence of etoposide usage in men for major indications such as acute myeloid leukemia (AML), acute lymphocytic leukemia, Hodgkin’s lymphoma, small cell lung cancer, and specific indications like testicular cancer (Sant et al., 2010; Townsend and Linch, 2012; Hellesøy et al., 2021; Rudin et al., 2021; Chovanec et al., 2023). Furthermore, our study highlighted that approximately 90% (88.7%) of adverse event reports were provided by health professionals, which adds credibility to the results of our analysis.

### 4.2 Blood and infection-related adverse reactions

Based on disproportionality analysis, we found that the most common and significant ADEs at the SOC levels included “blood and lymphatic system disorders,” and “infections and infestations”. One of the most frequent dose-limiting adverse reactions in cancer therapy is hematotoxicity (n = 87, ROR 23.16 [18.74–28.63]). The fast blood cell turnover renders them a potential target for conventional chemotherapy, and such toxicity can contribute to a range of blood disorders (Haglund et al., 2010). Furthermore, there were many previous clinical studies that also confirmed the hematological toxicity of etoposide. Tonder’s research suggested that 8 of 12 high-grade glioma patients occurred hematotoxicity of World Health Organization (WHO) grade 3 or 4 after administration of carboplatin and etoposide (Tonder et al., 2014). In another multicenter phase II trial in small cell lung cancer (SCLC), treatment with etoposide resulted in grade 3 to 4 leukopenia and grade 3 thrombocytopenia in 74% and 10% of patients, respectively (Bremnes et al., 2001). A meta-analysis bringing together the results of three randomized controlled trail in SCLC showed that etoposide/cisplatin was more likely to have blood-related side effects than irinotecan/cisplatin (Jeremić and Milićić, 2007). Through a real-world analysis of etoposide, we had also identified a number of significant hematologic adverse signals including anemia (n = 345, ROR 3.64 [3.27–4.04]), leukopenia (n = 176, ROR 7.36 [6.34–8.54]), thrombocytopenia (n = 417, ROR 7.96 [7.23–8.77]), and myelosuppression (n = 47, ROR 6.32 [4.74–8.41]), which were consistent with the results of previous clinical trials and the drug’s instructions. Furthermore, we have identified novel, unlabeled signals in the instructions, such as thrombotic microangiopathy (n = 35, ROR 8.41 [6.04–11.73]). Although the risk of thrombotic microangiopathy has been reported to increase significantly with etoposide after autologous stem cell transplantation in neuroblastoma patients, the specific role of etoposide in causing vascular endothelial injury requires further investigation (Vantelon et al., 2001; Jodele et al., 2018).

In addition to hematologic toxicity, various opportunistic infections are considered to be strongly associated with increased patient mortality, reduced chemotherapy doses, treatment delays, and increased healthcare costs (Peretz et al., 2016; Nordvig et al., 2018; Abdel-Azim et al., 2019). The reported incidence of infections associated with etoposide use in different clinical trials of SCLC ranged from 6% to 33% (Saito et al., 2006; Morabito et al., 2017; Socinski et al., 2017; Shimokawa et al., 2023). Additionally, similar infections have been reported in the treatment of tumors of blood, ovarian, prostate and breast origin (Dahl et al., 2000; Papandreou et al., 2002; Lu et al., 2015; Matsumoto et al., 2015). The myelosuppressive effect of etoposide, particularly its impact on neutrophil production, is likely the main contributing factor to the development of various aggressive infections. These infections, in turn, can further impair neutrophil production and hasten their depletion. Therefore, prophylactic administration of colony-stimulating factor injections is necessary (Urban et al., 1996; Kuderer et al., 2007; Mhaskar et al., 2014; Wang et al., 2015). In conclusion, it is imperative for clinicians to closely monitor patients’ coagulation function following the administration of etoposide. Antiplatelet agents should be used with caution, particularly in patients identified as high-risk for thrombosis during pre-treatment evaluation. Furthermore, timely intervention is crucial for managing various potential infections.

### 4.3 Neoplasms-related adverse reactions

At the neoplasm level, we also identified a number of ADEs with strong signal values. Second primary malignancy (SPM) (n = 43, ROR 10.32 [7.64–13.93]) is defined as a second, distinct pathological diagnosis of the same or different origin as the first primary malignancy, and chemotherapy also increases the risk of secondary hematologic or solid malignancies (Lenzi et al., 2020; Geng et al., 2023). The mechanism of etoposide-induced second primary malignancy (SPM) can be attributed to two possible explanations. Firstly, it can cause translocation rearrangement of the MLL gene on chromosome 11q23 (Ezoe, 2012). Secondly, the formation of catechol during drug metabolism can contribute to SPM development (Hartmann and Lipp, 2006; Zahnreich and Schmidberger, 2021). In addition, our study revealed that tumor lysis syndrome (TLS) also has strong signal value (n = 145, ROR 37.61). TLS occurs due to the rapid breakdown of malignant cells, leading to the release of cellular contents, such as electrolytes, nucleic acids, and metabolites, into the bloodstream. This phenomenon typically happens spontaneously or after treatment in patients with malignancies (Durani and Hogan, 2020; Grewal et al., 2023). TLS is characterized by its high lethality and is often associated with the administration of cytotoxic chemotherapy. Clinical manifestations of TLS primarily include hyperuricemia, hyperkalemia, and hyperphosphatemia, which can result in acute respiratory distress, disseminated intravascular coagulation, and renal failure (Calvo Villas, 2019; Tambaro and Wierda, 2020). We came across a case report which described a patient with testicular cancer who developed TLS and eventually succumbed to an infection and respiratory distress syndrome after receiving etoposide chemotherapy (Kobatake et al., 2015). It is therefore essential to assess the risk before chemotherapy and closely monitor electrolyte levels after treatment in patients with high-risk factors for TLS, such as those with highly proliferative hematologic tumors or pre-existing renal dysfunction (Durani and Hogan, 2020). In conclusion, our study alerts clinical decision makers that they should be aware of these lethal tumor-related signals during etoposide administration.

### 4.4 Adverse reactions at other SOC level

ADEs associated with etoposide administration may also involve other organs or tissues based on our disproportionality analysis. The observed cardiac disorders of etoposide use in our research included cardiotoxicity (n = 73, ROR 319.62 [15.57–24.71]) and acute myocardial infarction (n = 53, ROR 3.37 [2.58–4.42]).The metabolic disturbances caused by chemotherapy drugs and the oxidative damage by the oxygen radicals they produce might be reasonable explanations for its cardiotoxicity (Pai and Nahata, 2000; Simbre et al., 2005). As for the nephropathy toxic (n = 72, ROR 14.88 [11.79–18.77]) associated with etoposide administration, in addition to the above-mentioned TLS that might trigger renal failure, other possible explanations include delayed clearance of the drug in the kidney or an increased burden on the kidney from microthrombosis. Other than the more common adverse reactions listed above, there are a number of less commonly reported toxicities involving the ear and reproductive system that require caution. Ear and labyrinth disorders associated with etoposide administration were identified in our study including ototoxicity (n = 37, ROR 44.18 [31.86–61.25]) and deafness (n = 51, ROR 4.10 [3.12–5.40]), which may be related to drug-induced damage to cochlear hair cells (Kushner et al., 2006). Notably, an analysis of adverse drug reaction (ADR) reports describing drug-induced ototoxicity from the Italian spontaneous reporting system also identified a potential role for etoposide in the development of tinnitus, which is also in agreement with our results (Barbieri et al., 2019). Regarding the relationship between etoposide and ovarian failure (n = 43, ROR 179.93 [131.29–246.60]), it was reported that Anti-Muller hormone (an indicator of ovarian reserve function) was significantly lower in patients receiving etoposide-containing chemotherapy compared to the general population, and reduced ovarian function was difficult to restore after discontinuation of the drug (Meissner et al., 2015; Anderson et al., 2018). Prior to chemotherapy with etoposide, female patients should be informed of the potential gonadal toxicity. Age-specific discussions and fertility preservation procedures should also be considered, such as the use of gonadotropin-releasing hormone agonist prior to chemotherapy to reduce the number of primordial follicles entering the differentiation phase and to reduce follicular apoptosis, thereby protecting ovarian reserve function (Blumenfeld, 2007; Moore et al., 2015). In conclusion, the above newly identified signals in different organs may need to be specified in subsequent updates of the drug specification.

### 4.5 Time-to-onset and gender difference of ADEs

The findings of our study indicate that the majority of ADEs following etoposide treatment occur within 3 months, with the highest incidence observed in the first month (73.8%). In total, 89.4% of ADEs were reported within the first 3 months. Given this information, it is crucial to pay close attention to ADEs within the first month following etoposide administration. Timely identification and management of adverse events caused by etoposide therapy at an early stage are essential. It is noteworthy that there is a lack of comprehensive studies focusing on the specific timing of adverse reactions after etoposide administration, making our study a valuable contribution in this area. Gender differences have been shown to affect the bioavailability, distribution, metabolism, and elimination of drugs, leading to variations in ADEs between males and females (Zopf et al., 2009; de Vries et al., 2020; Farkouh et al., 2020). However, there is a lack of reported gender-specific ADEs associated with etoposide treatment. In our study, we observed that females had a higher number of positive signal values for ADEs compared to males. This finding aligns with previous research indicating that females are more prone to experiencing ADEs (Tran et al., 1998; Anderson, 2005). Interestingly, in males, we identified pneumonia as a high-risk signal, which may be attributed to their longer airways compared to females (Talaminos Barroso et al., 2018). Enhancing our understanding of gender-related ADEs will contribute to improving drug safety, efficacy, and optimizing drug therapy for both males and females (Sharifi et al., 2021). Subsequent clinical trials and mechanistic studies are necessary to validate and provide explanations for these ADEs with gender differences. This will guide better drug regimens for both males and females.

The present research, although suggesting a potentially significant relationship between the use of etoposide and the likelihood of reporting ADEs in FAERS, is not without limitations. First, it is important to acknowledge that FAERS is a spontaneous reporting system, and information collected from various countries and professionals may be incomplete or inaccurate, which can introduce bias into the analysis results. Second, despite our detailed explanation in the discussion section, FAERS alone cannot provide sufficient evidence to establish a causal relationship between drug use and ADEs (Shu et al., 2022c). Therefore, our findings should be viewed more as a warning to clinicians and pharmacists to remain vigilant regarding potential adverse events. Third, it is worth noting that monotherapy is uncommon in cancer treatment. Although etoposide was identified as the primary suspect in the reported adverse events in our analysis, it is challenging to determine the adverse effects solely caused by etoposide (Ezoe, 2012). Finally, it is also worth exploring how these ADEs impact across races, or across regions (Sabblah et al., 2017)? It is crucial to consider these limitations when interpreting the findings of our research and to encourage further investigations, including clinical trials and self-testing cohort data of clinical dosing information, to validate and expand upon our observations.

## 5 Conclusion

In conclusion, this study conducted a scientific and systematic analysis of adverse reactions linked to etoposide dosing, including their onset times and potential gender differences using the FAERS database. It is crucial that clinicians maintain a high level of vigilance regarding these potentially serious ADEs. Additionally, considering the potential gender differences is important for optimizing drug selection and closely monitoring patients. Further prospective clinical studies are required to confirm and enhance our understanding of the association between etoposide and these ADEs.

## Data Availability

The original contributions presented in the study are included in the article/Supplementary material, further inquiries can be directed to the corresponding author.
